# Diversity of the RFamide Peptide Family in Mollusks

**DOI:** 10.3389/fendo.2014.00178

**Published:** 2014-10-24

**Authors:** Celine Zatylny-Gaudin, Pascal Favrel

**Affiliations:** ^1^Université de Caen Basse-Normandie, Normandie Université, Biology of Aquatic Organisms and Ecosystems (BOREA), Caen, France; ^2^Muséum National d’Histoire Naturelle, Sorbonne Universités, BOREA, Paris, France; ^3^Université Pierre et Marie Curie, BOREA, Paris, France; ^4^UMR 7208 Centre National de la Recherche Scientifique, BOREA, Paris, France; ^5^IRD 207, L’Institut de recherche pour le développement, BOREA, Paris, France

**Keywords:** FaRPs, LFRFamide, luqin, NPF, CCK/SK, mollusks

## Abstract

Since the initial characterization of the cardioexcitatory peptide FMRFamide in the bivalve mollusk *Macrocallista nimbosa*, a great number of FMRFamide-like peptides (FLPs) have been identified in mollusks. FLPs were initially isolated and molecularly characterized in model mollusks using biochemical methods. The development of recombinant technologies and, more recently, of genomics has boosted knowledge on their diversity in various mollusk classes. Today, mollusk FLPs represent approximately 75 distinct RFamide peptides that appear to result from the expression of only five genes: the FMRFamide-related peptide gene, the LFRFamide gene, the luqin gene, the neuropeptide F gene, and the cholecystokinin/sulfakinin gene. FLPs display a complex spatiotemporal pattern of expression in the central and peripheral nervous system. Working as neurotransmitters, neuromodulators, or neurohormones, FLPs are involved in the control of a great variety of biological and physiological processes including cardiovascular regulation, osmoregulation, reproduction, digestion, and feeding behavior. From an evolutionary viewpoint, the major challenge will then logically concern the elucidation of the FLP repertoire of orphan mollusk classes and the way they are functionally related. In this respect, deciphering FLP signaling pathways by characterizing the specific receptors these peptides bind remains another exciting objective.

## Introduction

**Figure 1:**
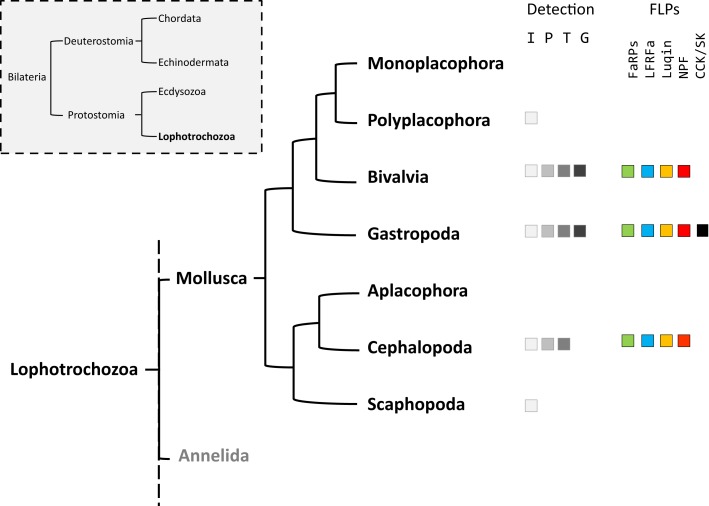
**Phylogenic tree of mollusks based on Stöger et al. (1)**. The occurrence of the different FMRFamide-like peptides (FLPs) in each class is detailed. F_a_, FMRFa-related peptides or FaRPs; L_r_, LFRFamide peptides; L_u_, Luqin; N_p_, NPF; C_ck_, cholecystokinin-/sulfakinin-like peptides. The methodologies used for FLPs detection are indicated: I, immunohistochemistry; P, peptide identification; T, transcript characterization; G, genome.

Mollusks exhibit great morphological diversity. They have adapted to marine, freshwater, and terrestrial habitats. They have distinct reproductive strategies; some species are gonochoric, simultaneous hermaphrodite, or alternative hermaphrodite. The phylum Mollusca is divided into eight classes (1): Monoplacophora (*Neopilina*), Polyplacophora (chitons), Bivalvia (clams, oysters, mussels), Gastropoda (snails, slugs), Aplacophora (worm-like mollusks), Cephalopoda (squid, cuttlefish, octopus), and Scaphopoda (tusk shells). Neuropeptides play a crucial neurotransmitter, neuromodulator, or neurohormone role in the elaboration of adapted physiological and behavioral responses to environmental constraints. As a result, phylogenetic distances, together with body plan and physiological behavior diversity probably reflect differences in the composition of mollusks’ neuropeptide record and their pattern of expression. The relatively simple central nervous system (CNS) of the gastropod mollusks *Aplysia californica* and *Lymnaea stagnalis*, with large identified neurons, has made them the most widely studied species for deciphering the role of neuropeptides in the control of physiological processes and behaviors (2–4). In mollusks, among the multiplicity of neuropeptides, the cardioexcitatory neuropeptide FMRFamide was first isolated in the clam *Macrocallista nimbosa* (5). So far, FMRFamide along with its structurally related neuropeptides or FMRFamide-like peptides (FLPs) that display varying sizes but harbor the common C-terminal RFamide sequence probably represent the best investigated family of neuropeptides in mollusks. Initial studies essentially concerned immunocytochemical screening of mollusk tissues with antibodies raised against the tetrapeptide FMRFamide. Then, with the refinement of peptide purification and sequencing methodologies, many new related peptides were discovered. By rendering the structure of the precursors encoding this family of peptides accessible, the development of recombinant technology has progressively extended knowledge about the diversity of FLPs. Knowledge includes FMRFamide-related peptides (FaRPs) and other RFamide peptides, especially in mollusks where purification was difficult for anatomical reasons (Figure 1).

In addition to mollusks (6), the FLP family has been extensively described in all major animal phyla, from cnidarians to mammals (7, 8), and are involved in the regulation of a great variety of physiological processes (9–12). With the advent of the genomics era, bioinformatics analyses of transcriptome and genome databases now clearly suggest that the high diversity of RFamide peptides in mollusks results from the expression of only five distinct genes: the FMRF gene, the LFRFamide gene, the luqin gene, the neuropeptide F (NPF) gene, and the cholecystokinin/sulfakinin (CCK/SK)-related gene (Figure 1).

## The FMRF Gene Codes for a Diversity of FMRFamide-Related Peptides

Since the discovery of the FMRFamide, a cardioactive neuropeptide in the clam *M. nimbosa* (5), an increasing number of studies has demonstrated the presence of this peptide and related peptides (FaRPs) in other mollusks (Table 1) and other protostomes [for reviews, see Ref. (6, 8)].

**Table 1:** **Diversity of FMRFamide-related peptides or FaRPs in mollusks**.

Peptide sequence	Species	Reference
FMRFa	*Macrocallista nimbosa*	(5)
pQDPFLRFa	*Helix aspersa*	(13)
AFLRFa	*Octopus vulgaris*	(14)
TFLRFa	*Octopus vulgaris*	(14)
FLRFa	*Lymnaea stagnalis*	(15)
SFMRFa	*Lymnaea stagnalis*	(15)
NDPFLRFa	*Helix aspersa*	(16)
NDPYLRFa	*Helix aspersa*	(16)
SEPYLRFa	*Helix aspersa*	(16)
GDPFLRFa	*Lymnaea stagnalis*	(17)
SDPFFRFa	*Lymnaea stagnalis*	(18)
SDPFLRFa	*Lymnaea stagnalis*	(17)
SDPYLRFa	*Lymnaea stagnalis*	(17)
pQFYRFa	*Helix aspersa*	(19)
ENNNGYIRFa	*Helix aspersa*	(19)
*SYGWAEGDTTDNEYLRFa	*Helix aspersa*	(19)
AdLAGDHFFRFa	*Mytilus edulis*	(20)
DPFLRFa	*Helix pomatia*	(21)
pQGDTADNEYLRFa	*Helix pomatia*	(21)
SKPYMRFa	*Lymnaea stagnalis*	(22)
ALTNDHELRFa	*Fusinus ferrugineus*	(23)
PYMRFa	*Lymnaea stagnalis*	(18)
HDYMRFa	*Lymnaea stagnalis*	(18)
FIRFa	*Sepia officinalis*	(24)
ALSGDAFLRFa	*Sepia officinalis*	(24)
*NFLRFa	*Mytilus edulis*	(25)
GPMGWVPVFYRFa	*Conus spurius*	(26)
*ALAGDGFLRFa	*Lottia gigantea*	(27)
NFGEPFLRFa	*Haliotis asinina*	(28)
TLAGDSFLRFa	*Haliotis asinina*	(28)
*FDSYEDKAYLRFa	*Haliotis asinina*	(28)

Peptide sequences: *, only predicted by cDNA or EST; a, amide.

Immunohistochemistry assays using an anti-FMRFamide serum have evidenced the expression of a diversity of RFamide-like peptides in the five classes of mollusks through a variety of nervous structures and peripheral tissues. After the initial studies demonstrating the widespread distribution of FMRF-amide immunoreactivity in perikarya and nerve fibers in the central and peripheral nervous system of the pond snail *L. stagnalis* (29, 30), presence of FMRFamide immunoreactivity was investigated in a significant collection of mollusks. In the bivalve *Placopecten magellanicus*, immunoreactivity was not only concentrated in the cerebral, pedal, and parietovisceral ganglia but was also equally localized in the peripheral organs, including the gut and gills of juveniles and adults (31). In *Dreissena polymorpha*, immunoreactivity was observed in all nervous ganglia and in the neuromuscular system associated to the siphon and mantle (32). In the gastropod *Helix aspersa*, immunoreactivity revealed a highly developed peripheral nervous system where nerves containing FMRFamide were associated to the muscular fibers of the reproductive or digestive tracts (33, 34). In the cephalopod *Sepia officinalis*, immunoreactive neurons were detected in the CNS at the olfactory and basal-dorsal lobe of the supra-esophageal brain mass as well as in the vicinity of the optic gland (35). In this species, FMRFamide-containing motoneurons innervating the fin chromatophore muscles are localized to the posterior chromatophore and fin lobes in the posterior subesophageal mass of the brain (36). The occurrence of FLPs during mollusk development was also observed in the sensory system of chiton larvae (polyplacophores) (37) or during the development of the cerebral system, the visceral loop, and the buccal nerve cords in scaphodod larvae (38). However, the use of FMRFamide antisera recognizing the antigenic major determinant RFamide likely covers the detection of different sets of peptides.

Different FLPs including FaRPs have been characterized by HPLC separation and amino acid sequencing by Edman degradation. Besides the tetrapeptide identified in several gastropod or cephalopod species, other peptides defined as FaRPs have been described; FaRPs include all the peptides with the C-terminal consensus sequence X_1×2_RFamide where X_1_ is an aromatic amino acid Phenylalanine (F), Tryptophane (W), or Tyrosine (Y) and X_2_ a hybrophobic amino acid Phenylalanine (F), Methionine (M), Isoleucine (I), or Leucine (L) (39). Thus, the pentapeptides AFLRFamide and TFLRFamide were identified from immunoreactive fractions of *Octopus vulgaris* vena cava (14). The heptapeptides SDPFLRFamide and GDPFLRFamide were identified from immunoreactive fractions in *L. stagnalis* (40) but also in *H. aspersa* where other heptapeptides (pQDPFLRFamide, NDPYLRFamide, and SEPYLRFamide) were isolated (13, 16). The first decapeptide (AdLAGDHFFRFamide) related to FaRPs was purified from *Mytilus edulis* based on its excitatory effect on the anterior byssus retractor muscle (ABRM) (20). This decapeptide has a d-leucine in position 2 that does not appear essential for biological activity of the peptide on ABRM (20). Surprisingly, in the venom of *Conus spurius*, two singular peptides inducing hyperactivity syndrome in mouse have been identified. The first one (GPMGWVPVFYRFamide) belongs to the FaRPs family (26), and the second one exhibits two gamma-carboxyglutamates and an IIRIa C-terminal sequence (GPMγDPLγIIRIa, with γ = gamma-carboxyglutamate) (41).

**Figure 2:**
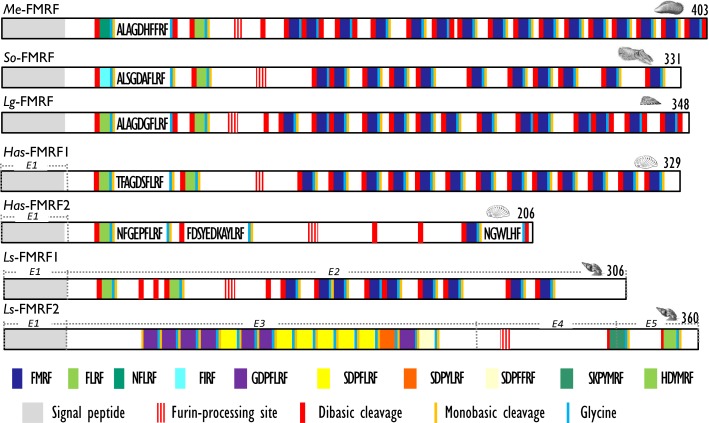
**Comparison of the linear schematic organization of FMRFa precursors in mollusks: *Me*-FMRF identified in *Mytilus edulis* (25), *So*-FMRF in *Sepia officinalis* (24), *Lg*-FMRF in *Lottia gigantea* (27), *Has*-FMRF1 and *Has*-FMRF2 in *Haliotis asinina* (28), and *Ls*-FMRF1 and *Ls*-FMRF2 in *Lymnaea stagnalis* (18)**. Signal peptides, proteolytic processing sites, and C-terminal glycines for amidation are indicated by distinct labels. For *Ls*-FMRF1 and *Ls*-FMRF2 precursors, the respective encoding exons are indicated as E1, E2, E3, E4, and E5.

With the characterization of cDNAs, the number of FaRPs identified in mollusks increased and gave a more rational basis for this diversity. The first mRNA encoding FaRP precursors was characterized in *A. californica* (42, 43) and revealed the occurrence of 28 copies of the FMRFamide sequence associated with a single copy of FLRFamide. Thereafter, several transcripts coding for multiple copies of the FMRFamide sequence were identified in other gastropods like *L. stagnalis* (17) and *H. aspersa* (19), in the bivalve *M. edulis* (25) and in cephalopods such as *S. officinalis* (24). In cuttlefish and squid, FMRFamide transcripts encode four identical FaRPs: three tetrapeptides (FMRFamide, FLRFamide, FIRFamide) and one decapeptide (ALSGDAFLRFamide) (44). In *O. vulgaris*, partial cDNA predicts the presence of the same ALSGDAFLRFamide decapeptide associated to an uncommon FMKFamide tetrapeptide (45). A common model of FMRFamide precursor can be defined based on the analysis of all of these tetrapeptide encoding transcripts (Figure 2; Table 2). It contains a unique tetrabasic site corresponding to a furin-processing site (RXK/RR) that typically separates the precursor into two domains: the N-terminal region encoding the FL/IRFamide peptides and a decapeptide, while the C-terminal domain harbors the FMRFamides. As a result, peptides from the two separate domains may presumably be sorted differentially into distinct secretory vesicles, as suggested for *L. stagnalis* FMRF gene products (46) and demonstrated for egg-laying prohormone gene products in *A. californica* (47) and *L. stagnalis* (48).

**Table 2:** **FaRP precursors from different species of mollusks with indication of copy numbers for each peptide category**.

Mollusk class	Transcript	Tetrapeptides			Pentapeptides	Hexapeptides	Heptapeptides	Non-apeptides	Decapeptides	Dodecapeptides	Heptadecapeptides	Reference
		FMRFa	FLRFa	FIRFa	
Gastropods	*Ac*-FMRF	28	1									(43)
	*Ls*-FMRF1	9	2									(18)
	*Ls*-FMRF2					1	16					(18)
	*Ha*-FMRF1	10	2		2							(19)
	*Ha*-FMRF2						13	1			1	(19)
	*Has*-FMRF1	13	2						1			(28)
	*Has*-FMRF2	1	1					1		1		(28)
	*Lg*-FMRF	14	2						1			(27)
Cephalopods	*So*-FMRF	11	1	1					1			(24)
	*Lo*-FMRF	11	1	1					1			(49)
	*Lp*-FMRF	11	1	1					1			(49)
	*Ov*-FMRF	7	1						1			(45)
Bivalves	*Me*-FMRF	16	2		1				1			(25)

*Ac, Aplysia californica; Ls, Lymnaea stagnalis; Ha, Helix aspersa; Has, Haliotis asinana; Lg, Lottia gigantea; So, Sepia officinalis; Lo, Loligo opalescens; Lp, Loligo pealei; Ov, Octopus vulgaris; Me: Mytilus edulis*.

There exists a second transcript coding for FaRPs in some gastropods. In the pulmonate mollusks *L. stagnalis* (17) and *H. aspersa* (19), this second mRNA mostly codes for heptapeptides (Table 2) and represents an alternatively spliced transcript that only shares the 5′exon with the tetrapeptide type transcript. In *L. stagnalis* (18), *Ls*-FMRF1 mRNA includes exons E1 and E2 and *Ls*-FMRF2 mRNA exons E1, E3, E4, and E5 (Figure 2). Singularly, two mRNAs were also characterized in the gastropod *Haliotis asinana*. These mRNAs share the first exon coding for the signal peptide and appear to be splice variants of a single gene, but in contrast to *Lymnaea* alternative transcripts they both encode copies of FMRFamide and FLRFamide along with a small number of new peptides. These two FaRP transcripts are differentially expressed in the gastropod nervous system. In adult *H. aspersa* snails, tetraFaRP mRNA (*Ha*-FMRF1) is located primarily in the cerebral ganglia, whereas heptaFaRP mRNA (*Ha*-FMRF2) is expressed almost exclusively in the parietal ganglia (50). In *L. stagnalis*, the two alternative mRNA transcripts are expressed in the CNS in a mutually exclusive manner at the single cell level; as a result, the distinct sets of FaRPs that they encode are differentially distributed in defined neuronal networks (51). FaRPs are expressed early during embryo development and larval development in mollusks with indirect development. In *Lymnaea* embryos, the differential localization of FaRPs could be established with specific antibodies against EFLRIa expressed by tetraFaRP mRNA (*Ls*-FMRF1) or against the acidic spacer peptide expressed by heptaFaRPs mRNA (*Ls*-FMRF2). Thus, the first neurons in *Lymnaea* embryos co-express the two transcript products, but neurons differentiate rapidly during ganglion development by expressing peptides of only one type of transcript. Otherwise, abundant EFLRIa-immunoreactive cells (corresponding to *Ls*-FMRF1) have been observed in the lip, mantle, and foot of larvae, but no peripheral cells immunoreactive to antibodies raised against the acidic peptide (corresponding to an *Ls*-FMRF2 cleaved product) have been found (52). *In H. asinina*, the two transcripts are always co-expressed in the larval ganglia but as in *Lymnaea* larvae, *Has*-FMRF1 is expressed alone in the periphery of putative sensory cells of the foot (28). In the cephalopod *S. officinalis*, expression of FaRP-encoding mRNAs was also observed during embryogenesis in the nervous system from the early steps of organogenesis (stage 16). Wider FaRP expression was observed concomitantly with brain differentiation (around stage 22) (53).

In mollusks, as in other invertebrates, FaRPS are involved in a variety of physiological processes including regulation of circulation, neuronal activity, feeding, digestion, reproduction, and osmoregulation. After the initial discovery of the excitatory effect of FMRFamide on the heart of the clam *M. nimbosa* (5), the cardioexcitatory or the cardioinhibitory effects of FaRPs on the cardiac muscle were later confirmed in other bivalves (54). In gastropods, FaRPs induced contractions of the ventricles by triggering calcium release from internal pools (55). Besides their direct activity on the heart, FaRPs also play a role in the circulatory system by acting on vessels, as shown in *A. californica* where the FMRFamide inhibited aorta contractions in synergy with serotonin (56). FaRPs are also involved in the control of the feeding behavior in mollusks, as observed in other invertebrates and for the other FLPs in vertebrates [for a review, see Ref. (57)]. The FMRFamide inhibited motoneurons B15 and B16 but excited interneurons B4 involved in the control of the feeding behavior in *A. californica* (58). FMRFamide affects the feeding system in other gastropods like *Lymnaea* and *Helisoma* where bath application of FMRFamide slowed down fictive feeding (59, 60). In *Lymnaea*, FMRFamide rapidly attenuated the rhythmic firing of buccal and cerebral ganglion neurons responsible for initiating and maintaining the repetitive motor outputs required for feeding (61). In addition, the tetrapeptide acts directly on muscles as described in *Aplysia*. FMRFamide modulate the activity of radula opener muscles in association with serotonin and myomodulins (62), and FaRPs play a role in digestion by modulating spontaneous gut activity or inducing contractions of the gizzard in *Aplysia* (63). In the gastropod *Helisoma*, the FMRFamide suppresses the activation of the salivary glands by directly acting on gland cells and on the effector neuron (neuron 4) (64). The effect on secretion activity was confirmed in scallop (*Pecten maximus)*, where the FMRFamide exhibited a secretagogue effect on α-amylase secretion from the stomach-digestive gland complex (65). The FaRPs widely expressed in the CNS modulate neuronal activity. FMRFamide has a powerful inhibitory influence on bag cell neurons in *A. californica* by altering the properties of ion currents involved in the generation of action potentials and in the control of the resting potential (66). As bag cell neurons control egg-laying behavior, this effect on neurons can modulate a main physiological function like reproduction. Similarly, in the mollusk *L. stagnalis*, FMRFamide activates K+ currents that induce the inhibition of the caudodorsal neurons involved in the release of CDCH, the egg-laying hormone (ELH) (67). In cephalopods, FaRPs also appear to be involved in the control of reproduction regulation processes. In *Octopus*, the FMRFamide inhibits the activity of the optic gland involved in sexual maturity (45), whereas in cuttlefish, FaRPs control egg laying by directly regulating oocyte transport through the oviduct; the tetrapeptides FMRFamide and FLRFamide stimulate oviduct contractions, whereas FIRFamide and ALSGDAFLRFamide lower the tonus, the frequency, and the amplitude of the contractions and thus modulate them (68). FaRPs are also involved in specific functions in cephalopods. FMRFamide induces the contraction of chromatophore muscles involved in the control of body coloration patterning (69). The establishment of neural networks at the origin of this control was monitored during cuttlefish embryogenesis. FaRP staining throughout CNS development evidenced the implementation of neuronal networks involved in the control of coloration patterns. This suggests that the involvement of FaRPs in the chromatophore control pathway takes place early during embryonic development (53). In *Helisoma trivolvis*, the level of FaRPs in kidney, detected by radioimmunoassay, appeared to be lower in snails kept under hypoosmotic stress than in snails kept under isosmotic conditions, suggesting an involvement in gastropod osmoregulation (70).

FMRFamide-related peptides act synergistically with other mollusk-specific neuropeptides like APGWamide-related peptides. In *H. aspersa*, some cells contain both the APGWamide and FMRFamide; these cells may have dual projections in both the penial nerve and the nervus cutaneous pedalis primus dexter (71). In *Lymnaea*, different FaRPs and APGWamide have distinct actions on the penis retractor muscle; this demonstrates a complex peptidergic regulation of the male copulation behavior (72, 73). Together, these two tetrapeptides also modulate the feeding behavior in the gastropod mollusks *H. trivolvis* and *L. stagnalis* in association with other factors like biogenic amines (serotonin, dopamine, octopamine) (74). In the bivalve *M. edulis*, they act in synergy to modulate the contractile activity of the ABRM (75).

In mollusks, FaRPs appear to act via FMRFamide-gated sodium channels (FaNaC) and via G protein-coupled receptors. The first FaNaC was molecularly characterized in *H. aspersa* (76). Only gastropod FaNaCs have been characterized so far (77–79). FaNaC is a neuronal Na^+^-selective channel that is directly gated by micromolar concentrations of FMRFamide and related tetrapeptides inducing a fast and partially desensitizing response, and it is thought to participate in peptidergic neurotransmission. The sensitivity of *L. stagnalis* FaNaC to acids suggests common ancestry with mammalian acid-sensing ion channels (ASICs) (80), strengthened by the study of Askwith and collaborators concerning the direct modulation of rat ASICs by FMRFamide (81). Among the variety of candidate G-protein-coupled receptors (GPCRs) encoded by mollusk genomes, no FaRP-specific GPCR has been functionally characterized yet. Receptor-binding assays in *H. aspersa* suggest the presence of two receptors, one for tetrapeptides and another for heptapeptides localized in the heart, the brain, the reproductive, and digestive systems (82, 83). In a structure–activity relationship study of FMRFmide analogs in *Lymnaea*, the occurrence of a single receptor was proposed and demonstrated that activation of this receptor requires the C-terminal RFamide sequence, whereas the N-terminal amino acids are involved in binding. This unique receptor mediates the transient hyperpolarizing response and the long-lasting depression of excitability of the neurosecretory caudo-dorsal cells (CDCs) (84). In the squid, *Loligo pealei*, a radio receptor assay stressed the critical role of the RFamide moiety for binding (85).

## The LFRFamide/Short Neuropeptide F-Related Gene

LFRFamide peptides (Table 3) have been identified later. The first heptapeptides GSLFRFamide and SSLFRFamide were discovered from their inhibitory activity on F2 neurons of the prosobranch gastropod *Fusinus ferrugineus* (23). In parallel, in the opisthobranch *A. californica*, GSLFRFamide peptides were characterized, along with two other LFRFamide peptides with the STLFRFamide and GGALFRFamide amino acid sequences, from their activities on the accessory radula closer (ARC) neuromuscular system (86).

**Table 3:** **LFRFamide-related peptides**.

Peptide sequence	Species	Reference
GSLFRFa	*F. ferrugineus*	(23)
	*A. californica*	(86)
	*C. gigas*	(87)
SSLFRFa	*Fusinus ferrugineus*	(23)
	*C. gigas*	(87)
STLFRFa	*A. californica*	(86)
GGALFRFa	*A. californica*	(86)
GGSLFRFa	*Lymnaea stagnalis*	(88)
GTLLRFGa	*Lymnaea stagnalis*	(88)
NTLFRFGa	*Lymnaea stagnalis*	(88)
QGSLFRFGa	*Lymnaea stagnalis*	(88)
TLFRFa	*L. stagnalis, A. californica*	(88, 89)
GAGTLFRFa	*Aplysia californica*	(89)
GNLFRFa	*Sepia officinalis*	(90)
TIFRFa	*Sepia officinalis*	(90)
*NSLFRFa	*Sepia officinalis*	(91)
*PHTPFRFa	*Sepia officinalis*	(91)
GALFRFa	*Crassostrea gigas*	(87)
SVDNEPTHPFRFa	*Crassostrea gigas*	(87)

Peptide sequences: *, only predicted by cDNA or EST; a, amide.

**Figure 3:**
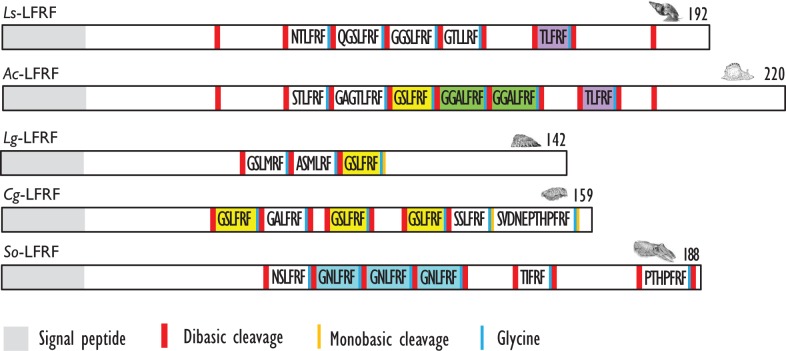
**Comparison of the linear schematic organization of LFRFa precursors in mollusks: *Ls*-LFRF identified in *Lymnaea stagnalis* (88), *Ac*-LFRF in *Aplysia californica* (86), *Lg*-LFRF in *Lottia gigantea* (27), *Cg*-LFRF in *Crassostrea gigas* (87), and So-LFRF in *Sepia officinalis* (91)**. Signal peptides, proteolytic processing sites, and C-terminal glycines for amidation are indicated by distinct labels.

For a long time, LFRFamide peptides were found only in gastropods, yet recent studies in cephalopods and oysters have evidenced this peptide family in other mollusk classes. In the cephalopod *S. officinalis*, two LFRFamide peptides (GNLFRFamide and TIFRFamide) were clearly characterized from LC-MS/MS analysis, with an experimental strategy based on the presence of the RFamide moiety (90). In oyster (*Crassostrea gigas)*, the LFRF precursor was retrieved from the GigasDatabase [CU994925] (92). The first LFRFamide precursor was identified in the pulmonate snail *L. stagnalis* from its overexpression following parasitization by *Trichobilharzia ocellata* (93). The precursor contains one of several types of LFRFamide peptides in *L. stagnalis* (88) and *Lottia gigantea* (27) (Figure 3). Sometimes, several copies of the same peptide are present as in the case of the LFRF precursor (*Ac*-LFRF) isolated from *A. californica*. In the *Ac*-LFRF precursor, two copies of the GGALFRFamide are associated with one copy of the GSLFRFamide, TLFRFamide, STLFRFamide, and GAGTLFRFamide sequences (89) (Figure 3).

The characterization of the *S. officinalis So*-LFRFamide precursor revealed the presence of four different LFRF peptides with three copies of the GNLFRFamide associated to one copy of the TIRFamide, NSLRFamide, and PHTPFRFamide sequences (91) (Figure 3). The recent identification of LFRFamide peptides in the oyster *C. gigas* (87) confirms the widespread distribution of this peptide family in the mollusk phylum, with two of the bivalve peptides (GSLFRFamide and SSLFRFamide) previously identified in gastropods, particularly in *F. ferrugineus*. Similar to the *So*-LFRF precursor, the *Cg* precursor contains three copies of the GSLFRFamide peptide associated to one copy of the SSLFRFamide, GAFLRFamide, and SVDNEPTHPFRFamide sequences (Figure 3). That last sequence is located in the C-terminal moiety of the *Cg*-LFRFamide precursor and shares a common sequence with the last peptide (THPFRFamide) of the *So*-LFRFamide precursor.

Similar to FaRPs, LFRFamide peptides are involved in the regulation of main physiological functions. Studies on the localization of LFRFamide transcripts in *L. stagnalis* during parasitization show an expression in neurons in the buccal ganglia and in the cerebral ganglia and suggest an involvement in feeding and reproduction (88). Schistosome parasites adjust the physiology and behavior of their intermediate molluscan hosts to their own benefit by altering the expression of regulatory neuropeptides. The very close presence of LFRFamide-expressing neurons to *Lymnaea* CDCs and light green cells (LGCs), two cell clusters that regulate female reproduction, and growth and metabolism, respectively, together with the inhibitory activity of FLRFamide peptides on these cells suggest that the parasite induces LFRFamide gene expression to suppress host metabolism and reproduction (88). In cuttlefish, the myotropic activity of the GNLFRFamide peptide differs from that of FaRPs. Only the contractions of the rectum were modulated by this heptapeptide, but those of the oviduct were not, suggesting a role only in digestion-associated processes but not in reproduction (90). *In situ* hybridization (ISH) carried out in all parts of the cuttlefish CNS confirmed the involvement of LFRFamide peptides in feeding but also in learning and memory and in the control of body patterning (94, 95).

A recent study aimed at the functional characterization of a *C. gigas* short NPF like receptor (Cg-sNPFR-like) identified oyster LFRFamide family peptides as specific ligands (87). The *Cg*-sNPFR-like receptor was more abundantly expressed in the ganglia of females compared to males, and upregulated in starved oysters suggesting a role in reproduction and feeding. In the gonad area, highest receptor gene expression was observed at the start of gametogenesis, when glycogen storage activity is maximal. Oyster LFRFamide peptides are thought to play a role in the coordination of nutrition, energy storage, and metabolism through the *Cg*-sNPFR-like receptor by promoting storage at the expense of reproduction. The involvement of LFRFa in feeding and learning is very similar to sNPF in insects (96–98), further suggesting ancestral relationship The authors propose that mollusk FLRFamide peptides could represent functional orthologs of insect sNPFs (87).

## The Luqin Gene

**Figure 4:**
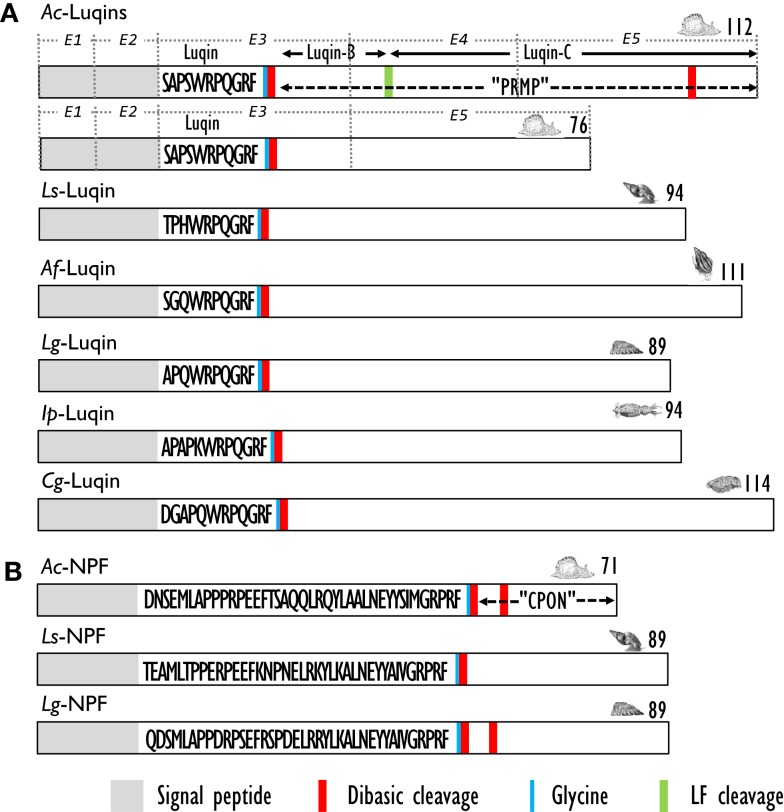
**(A)** Comparison of the linear schematic organization of Luqin precursors in mollusks: *Ac*-Luqin identified in *Aplysia californica* (101)*, Ls-*Luqin (LyCEP: *Lymnaea* CardioExcitatory Peptide) in *Lymnaea stagnalis* (104)*, Af-*Luqin (ACEP: *Achatina* CardioExcitatory Peptide) in *Achatina fulica* (108), *Lg-*Luqin in *Lottia gigantea*, *Ip*-Luqin in *Idiosepius paradoxus*, and *Cg*-Luqin in *Crassostrea gigas* (27). **(B)** Comparison of the linear schematic organization of NPF precursors in gastropods: *Ac*-NPF in *A. californica* (109), *Ls*-NPF in *L. stagnalis* (93), and *Lg*-NPF in *Lottia gigantea* (27). Signal peptides, proteolytic processing sites, and C-terminal glycines for amidation are indicated by distinct labels. CPON, carboxy-terminal peptide of NPF; PRMP, proline-rich mature peptide. For *Ac*-Luqin precursors, the respective encoding exons are indicated as E1, E2, E3, E4, and E5.

Using a differential screening strategy devoted to characterizing the neuropeptide-encoding transcripts specifically expressed in *A. californica* RF-amide immunoreactive L5 (left abdominal hemiganglion) neuron, a first precursor was characterized. L5 peptide precursor sequence predicts an RFamide decapeptide generated by signal peptide cleavage and by proteolytic cleavages at the Lys-Arg sequence. The C-terminal glycine of the resulting peptide is subsequently enzymatically converted into an amide group (99). A biochemical investigation showed that the processing of this precursor generates Luqin, a mature amidated decapeptide, together with a C-terminal proline-rich mature peptide (PRMP) of 76 amino acids (100). That latter peptide is subsequently proteolytically processed to generate two products, Luqin-B and Luqin-C (101) (Figure 4A). Alternative splicing by exon skipping of the gene encoding this precursor results in the production of a shorter PRMP with a distinct C-terminal sequence due to a reading frame shift (102). The *A. californica* decapeptide was named Luqin because it is differentially expressed in LUQ (left upper quadrant) neurons. Luqin displays a high degree of identity with the formerly identified cardioexcitatory undecapeptide ACEP1 isolated from atria of the African giant snail *Achatina fulica* (103) and with LyCEP, a decapeptide from *L. stagnalis* CNS isolated from its ability to activate an orphan GPCR (104). In addition to sequences from gastropod mollusks such as *L. gigantea*, expressed sequence tags (ESTs) encoding Luqin precursors were also identified from bivalve and cephalopod mollusks that potentially yield structurally highly conserved mature luqin peptides with only the first three amino acids exhibiting conservative substitutions (27) (Figure 4A). Interestingly, annelids also express Luqin precursors with an analogous organization and a predicted mature peptide of very similar structure (105) (Table 4). Recent studies on the evolution of neuropeptide-signaling components in animals suggest the ancestral presence of Luqin in bilaterians. Luqin has been proposed to have evolutionary links with ecdyzoan RYamide and ambulacrian RWamide precursors, as they all harbor a proline-rich C-terminal peptide displaying a conserved domain including two cysteine residues (106, 107).

**Table 4:** **Luqin-like peptides**.

Peptide sequence	Species	Reference
SGQSWRPQGRFa	*Achatina fulica*	(103)
SAPSWRPQGRFa	*Aplysia californica*	(100)
TPHWRPQGRFa	*Lymnaea stagnalis*	(104)
*APQWRPQGRFa	*Lottia gigantea*	(27)
*APAPKWRPQGRFa	*Idiosepius paradoxus*	(27)
*DGAPQWRPQGRFa	*Crassostrea gigas*	(27)

Peptide sequences: *, only predicted by cDNA or EST; a, amide.

Luqin was initially known to enhance the amplitude of the tetanic contraction of the heart ventricle, induce the tetanic contraction of the penis retractor muscle in response to electrical stimulation *in A. fulica*, or enhance the phasic contraction of the ABRM produced by repetitive electrical stimulation in *Mytilus* (103). Besides its activity as myoactive or cardioactive peptide, Luqin functional involvement was also inferred from the pattern of expression of peptide precursors or receptor transcripts by ISH or by immunocytochemical detection (ICC) of mature Luqin peptides using specific antibodies.

Luqin-encoding transcripts or mature Luqin peptides appear to be distributed in each of the central ganglia in around 20 neurons in *A. fulica* (108) and approximately 100 neurons including LUQ cells in *A*. *californica* (110). In *L. stagnalis*, prominent immunoreactivity was mainly found in neurons in the pedal ganglia (104). In this species, the caudodorsal cells that produce ELHs are the predominant site of Luqin- (LyCEP-) specific receptor gene expression and appear to be innervated by LyCEP-containing fibers involved in their inhibition (104). Thus, Luqin seems to inhibit the egg-laying system in that snail. In *L. stagnalis* peripheral tissues, Luqin immunoreactivity was detected in fibers ending blindly at the pericardial lumen (104). In *A. californica*, Luqin distribution was consistent with each LUQ cell sending its major axon through the pericardial nerve to innervate the kidney and the renal pore, a sphincter that controls urine efflux (111). Immunoreactive fibers were detected in specific regions of the circulatory system, such as the auricle, the ventricle, the cristae, and the anterior aorta, in the reproductive system (especially in the genital ganglion), the large and small hermaphrodite ducts, and the ovotestis (110). Intestine and kidney also display immunoreactivity in widely ramifying varicose fibers (110). In kidney, both Luqin transcripts and gene products were detected in neurites located in a large nerve associated with muscles inside the renal pore (112). Besides the conventional transcript, an alternatively spliced Luqin transcript appears to be specifically transcribed in peripheral neurons located in or in the vicinity of the kidney. Its gene products may locally play a physiological role by complementing or antagonizing the action of Luqin/PRMP peptides (102). Altogether, these data suggest a role of Luqin in the control of reproduction. Given the involvement of the target organs in fluid mobilization, absorption, and secretion, Luqin is also probably involved in the regulation of water and/or salt balance in mollusks.

## The Neuropeptide F (NPF) Gene

The first biochemical evidence for mollusk neuropeptide Y (NPY) homologs was provided by the amino acid sequence of a peptide isolated by means of a radioimmunoassay using an antibody raised against the C-terminal hexapeptide amide (LTRPRYamide) of mammalian pancreatic polypeptide (PP), in extracts of circumesophageal ganglia of the garden snail, *H. aspersa* (113). The 39 amino acid *Helix* peptide displayed significant homology with the vertebrate NPY/PP superfamily and with the first characterized invertebrate NPY homolog identified from the tapeworm *Monieza expansa* (114). Similar to its flatworm relative, the *Helix* C-terminal tyrosine-amide peptide (Ya) is substituted for a phenylalanine-amide peptide (Fa), so it was called NPF. Soon after the initial characterization of *Helix* NPF, *A. californica* NPF, a 40 amino acid long peptide, was isolated from the abdominal ganglia by using its distinctive, prolonged inhibitory effect on neurons L3 and L6. Next, the cDNA encoding its precursor protein was cloned and sequenced (115). *A. californica* NPF prohormone revealed an organization similar to vertebrate NPY precursor proteins; it had a single copy of NPF immediately following the signal peptide, and separated from the carboxy-terminal peptide of NPF (CPON) by a GKR sequence serving as combined amidation and prohormone convertase proteolytic signals (Figure 4B). At the same time, a nonapeptide with high homology with the C-terminal end of the other molluscan NPFs was purified from brain extracts of squid (*Loligo vulgaris*) (116). This peptide may represent a processed form of an authentic NPF peptide whose N-terminally truncated forms were also later characterized in other animal phyla [for a review, see Ref. (117)]. In *L. stagnalis*, a partial cDNA encoding an NPF prohormone was cloned by differential screening of a CNS-specific cDNA library of snails parasitized with *T. ocellata* (93) (Figure 4B). Then, the mature NPF peptide was structurally characterized after its purification to homogeneity using a reverse endocrinology approach. This method is based on the ability of fractionated brain extracts to induce changes in cAMP levels following the activation of an *L. stagnalis* orphan GPCR (GRL105) expressed in CHO cells (118). This receptor shares structural and functional similarity with vertebrate NPY-receptors and currently represents the only functionally characterized NPF receptor in mollusks. That a typical NPF could be isolated from the venom of the cone snail *Conus betulinus* (119) was somewhat intriguing though neuroendocrine-like peptides used for prey catching or defense have already been described (120). Completion of mollusk genome and transcriptome sequencing projects (91, 92) has now paved the way toward the identification of novel NPFs and their putative receptors. NPF members from owl limpet *L. gigantea* (27), pygmy squid *Idiosepius paradoxus* (121), and Pacific oyster *C. gigas* (87) have been identified (Table 5). In *C. gigas*, bioinformatics investigation of genome and transcriptome databases yielded two additional NPY sequences with a C-terminal tyrosine-amide instead of phenylalanine-amide (personal communication). All mollusk NPFs display a high degree of identity, especially among the last 20 C-terminal residues. They also have a conserved N-terminal pattern of proline residues probably involved in the formation of a polyproline helix proved essential for the three-dimensional structure of vertebrate NPYs (122).

**Table 5:** **NPF-related peptides**.

Peptide sequence	Species	Reference
YAIVARPRFa	*Loligo vulgaris*	(116)
STQMLSPPERPREFRHPNELRQYLKELNEYYAIMGRTRFa	*Helix aspersa*	(113)
DNSEMLAPPPRPEEFTSAQQLRQYLAALNEYYSIMGRPRFa	*Aplysia californica*	(115)
TEAMLTPPERPEEFKNPNELRKYLKALNEYYAIVGRPRFa	*Lymnaea stagnalis*	(118)
*MFAPPNRPAEFKSPEELRQYMKALNEYYAIVGRPRFa	*Idiosepius paradoxus*	(121)
*QDSMLAPPDRPSEFRSPDELRRYLKALNEYYAIVGRPRFa	*Lottia gigantea*	(27)
*NDSLLPPNRPSRFSSPGQLRQYLKALNDYYAIVGRPRFa	*Crassostrea gigas*	(87)

Peptide sequences: *, only predicted by cDNA or EST; a, amide.

Expression of NPF in these different species was investigated by ISH, mass spectrometry, and ICC using antibodies specific to each mollusk NPF or antibodies raised against vertebrate NPY/PP peptides, though in this later case, a cross-reaction with other FLPs cannot be discarded.

In *L. stagnalis*, ISH and whole-mount ICC studies revealed neurons expressing the NPF gene and peptide in all ganglia of the CNS, except the two buccal ganglia that innervate the buccal mass. In the visceral ganglion, one NPY-positive neuron projects its axon into the nervus intestinalis, which also innervates the reproductive tract including the accessory sex glands. In the CNS, NPF-positive axons run as a circular band through all ganglia passing the commissures connecting the ganglia. A close association was also found between NPF-positive axons and axons from ovulation hormone-producing neurons and molluscan insulin-like peptide-producing neurons involved in the regulation of growth (123). A subset of anterior lobe neurons expressing the gene encoding the APGWamide peptide was also shown to co-express NPF mRNA and peptide (124). These cells project via the penis nerve to the penial complex and play an important role in the control of male copulating behavior. NPF immunoreactivity was also detected in axons innervating the penis retractor muscle (124).

*Aplysia californica* NPF-encoding mRNAs are also widely expressed in the CNS, very abundantly in the abdominal ganglion, to a lesser extent in the pleural-pedal ganglia, and at much lower levels in the cerebral ganglion. In the abdominal ganglion, *A. californica* NPF coexists with ELH and other peptides processed from its precursor in bag cell neurons involved in the control of egg-laying behavior (115). In a study investigating the role of NPF in the control of feeding, whole-mount ISH and immunocytochemistry localized NPF mRNAs and peptides in several cerebral somata and in only one buccal neuron in the *A. californica* feeding system (109). NPF was present in fibers in all major connectives and especially in buccal ganglion afferent fibers originating in the gut-innervating esophageal nerve, a nerve involved in satiation and in the generation of egestive programs (109).

The widespread distribution of NPF immunoreactivity in the CNS and in a variety of target organs of *H. aspersa* was also demonstrated by ICC using antibodies specific to Helix NPF or vertebrate NPY (125). In *O. vulgaris*, ICC studies evidenced NPF immunoreactivity in the optic lobe and peduncle complex. In the optic gland, an endocrine organ that controls gonad maturation (126), NPF was detected in varicose fibers, suggesting a role in optic gland regulation (127).

The widespread and high expression of NPF in the CNS and the variety of target cells and organs innervated by NPF-containing fibers in mollusks emphasize the involvement of NPF signaling in the control of a number of physiological processes. In mollusks, the biological function of NPF has mainly been investigated in the two model species *L. stagnalis* and *A. californica*.

In *L. stagnalis*, the gene encoding NPF is upregulated in *T. ocellata*-parasitized animals (93). A role of NPF in the control of the energy flow was thus assumed. Indeed, parasites selectively interfere with neuroendocrine mechanisms that regulate the main determinants of the energy budget in the host’s reproduction and growth processes (128). NPF injection or implantation of slow-release peptide pellets in non-parasitized snails resulted in suppressed reproductive activity and reduced growth in a dose- and time-dependent manner (123). This activity appeared reminiscent of the well-known role of NPY in the regulation of the energy flow in vertebrates (129) but singularly differed by not affecting food intake (130). By contrast, NPF injection produced satiation-like effects in *A. californica*, i.e., a reduced meal-size and slower ingestion. NPF released from the esophageal nerve affects the feeding behavior; it modifies the feeding central pattern generator, a neuron network in the buccal ganglion, by eliciting egestive responses (109). Altogether, the distribution and the activity of NPF in mollusks point to a role in the coordination of the energy balance via the control of energy-consuming processes like growth and reproduction or possibly via the control of food intake. An NPF-related receptor-encoding mRNA was recently found significantly upregulated in oyster (*C. gigas*) lines with reduced reproductive investment and increased potential for energy production (131). Although care should be paid until the actual ligand of this receptor is identified, this result also suggests the control of NPF signaling in bivalve mollusk reproduction and energy management.

## The Cholecystokinin/Sulfakinin-Related Gene

A recent extended comparative survey of peptide signaling systems in bilaterians demonstrated the early evolutionary origin of the CCK/SK signaling system (106). In this study, *in silico* data-mining of *Haliotis diversicolor, A. californica*, *and L. gigantea* sequence resources revealed the existence of peptides with the following consensus sequence: X(1-6)(D/E)Y(G/N)(L/F/I)GGGR(F/W)-amide. These mollusk CCK/SK peptides exhibit the C-terminal RF(W)amide sequence common to insect SKs and the DY motif shared by both insect SKs and vertebrate CCKs (Table 6). Whether mollusk peptides are sulfated remains to be investigated. As vertebrate CCKs and insect SKs reveal similar biological functions with respect to digestive enzyme secretion, satiety (food intake), and smooth muscle contraction (132), we could expect their mollusk counterparts to have retained these basic biological activities. In this respect, it is interesting to note that FMRFamide and FLPs, including insect SKs, elicit potent stimulation of the release of the digestive enzyme α-amylase from cell suspensions of the stomach-digestive gland complex of scallop (*P. maximus*) (65, 133, 134).

**Table 6:** **CCK/SK-like peptides**.

Peptide sequence	Species	Reference
*FDYNFGGGRWa	*Lottia gigantea*	(106)
*QGAWDYDYGLGGGRFa	
NYGEYGFGGGRFa	*Haliotis diversicolor*	(106)
*QGAWSYDYGLGGGRFa	
*SYGDYGIGGGRFa	*Aplysia californica*	(106)
*QGAWSYDYGLGGGRFa		

Peptide sequences: *, only predicted by cDNA or EST; a, amide.

## Conclusion

**Figure 5:**
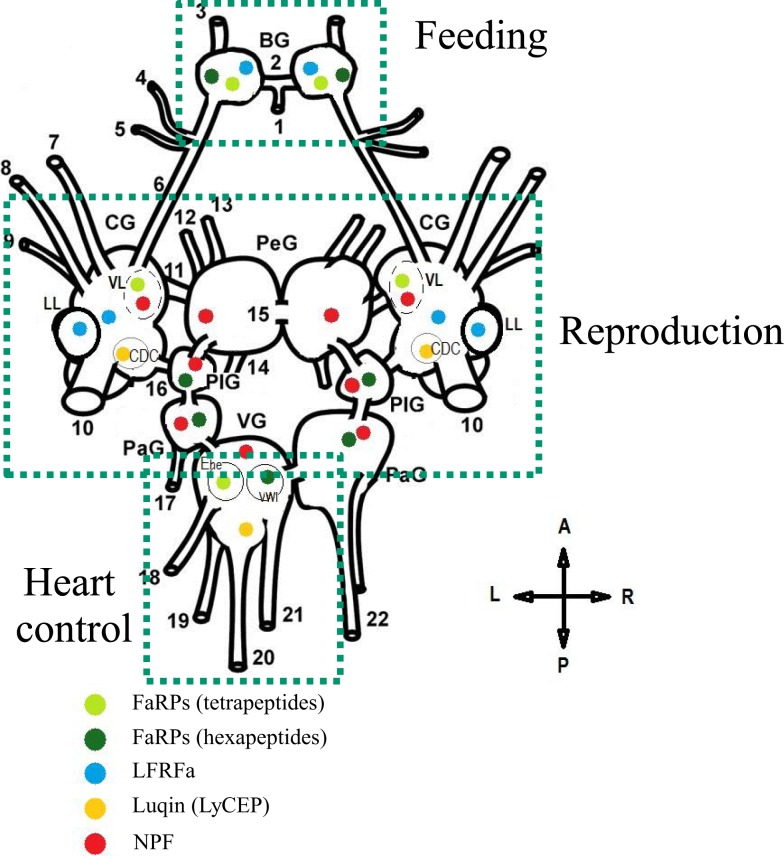
**Schematic representation of the distribution and the role of distinct RFamide-like peptides (FLPs) in the central nervous system (dorsal view) of *Lymnaea stagnalis***. BG, buccal ganglion; CG, cerebral ganglion; PeG, pedal ganglion; PlG, pleural ganglion; PaG, parietal ganglion; VG, visceral ganglion; VL, ventral lobe; LL, lateral lobe; CDC, caudo-dorsal cell. Nerves: 1, postbuccal; 2, buccal commissure; 3, dorsobuccal; 4, laterobuccal; 5, ventrobuccal; 6, cerebro-buccal connective; 7, superior lip; 8, median lip; 9, tentacular; 10, cerebral commissure; 11, cerebro-pedal connective; 12, superior medial; 13, median pedal; 14, inferior pedal; 15, pedal commissure; 16, cerebro-pleural connective; 17, left parietal; 18, cutaneous; 19, anal; 20, intestinal; 21, genital; 22, right parietal. Ehe neuron and the visceral white interneuron (VWI) [Adapted from Ref. (72, 104, 130, 141, 142)].

FMRFamide-like peptides exert pleiotropic activities and mediate a variety of physiological and behavioral processes in mollusks (Table 7). Interestingly, mollusks with diverse and well-defined habitats, life styles, and behaviors do not seem to exhibit significantly marked complexity levels in their FLP repertoires. This probably means that behavioral variety and adaptation to environmental cues result not only from the structural diversity of neuropeptide components but also from the intricacy of the spatiotemporal and stimulus-dependent patterns of expression of their encoding genes. Most of the functional assignments of this family of neuropeptides were obtained from the key model species *A. californica* and *L. stagnalis* because they have a CNS with large and reproducibly identified neurons, readily accessible and amenable to *in vivo* or *in vitro* handling. Since the activity of these neurons can also be correlated with physiological states or behaviors, establishing functional circuitries has become feasible in these species (61, 109, 135). As a result, a specific neuropeptide distribution within the CNS strongly infers its physiological involvement (Figure 5). Although sequence information is still lacking about so far completely understudied mollusk classes, such as monoplacophora, placophora, aplacophora, and scaphopoda, substantial genomic data are now becoming available in economically important classes of mollusks such as bivalves (91, 92) and cephalopods (136, 137). Such data offer the opportunity to investigate new mollusk models. These model species often have a compact and tiny CNS, so the methodologies developed for *A. californica* and *L. stagnalis* do not apply. Therefore, access to the neuropeptide-encoding gene repertoire of these non-conventional model species will necessarily require the improvement of currently operating post-genomic functional tools such as RNA interference (138) to succeed in deciphering their biological function.

**Table 7:** **Functional involvement of RFamide-like peptides in the three main mollusk classes: B, Bivalves; C, Cephalopods; G, Gastropods**.

	FaRPs		LFRF	Luqin	NPF
	Tetrapeptides	Hexapeptides	
Circulation/heart activity	B (5, 54)	G (16, 40, 55)		G (103, 104)	
	G (40, 55, 139)	
Feeding	G (58–62, 74)		G (86, 88)		G (109)
			C (94)	
			B (87)	
Digestion	G (63, 64)	G (64)	C (90)		
	B (65)	
Reproduction	G (34, 72, 84, 140)	G (34, 72)	G (88)	G (103, 104, 110)	G (115, 123, 124)
	C (45, 68)		B (87)	
Osmoregulation	G (70)	G (70)		G (102)	
Neuronal activity	G (66, 67)			G (110–112)	
Chromatophore activity	C (69)		C (95)		
Energy balance			B (87)		G (128)

In the field of neuroendocrinology, sequence data will become crucial for unraveling the complexity of signaling systems in an evolutionary context; genome-wide analysis gives access to the complexity of the repertoire of GPCRs, which constitute the main molecular targets of neuropeptides. In parallel, exhaustive interaction between bioinformatics and mass spectrometry analyses makes the molecular identification of most mature neuropeptides produced in a given species accessible (143). In the context of FLPs, gaining insight into the structure of the mature peptides appears crucial. Indeed, some peptides like CCK/SKs might be subjected to post-translational sulfatation. Others, like NPFs, are presumed to produce distinct truncated forms, with potentially distinct biological properties that derive from the non-conventional processing of a precursor protein (116, 144). The grouping of RFamide peptides within a same family might be somewhat misleading and restrictive, especially when considering the evolutionary links between the deuterostome and protostome NPY/F signaling systems, or between the Luqin and insect RYamide signaling systems (106, 107). Phenylalanine and tyrosine display very close physicochemical properties and the shift from one residue to the other only results from a single nucleotide mutation. We suggest that mollusk neuropeptides with the C-terminal RYamide might be included. These include the newly LFRYamide peptide family identified in *Lottia* (27) or neuropeptide KY (NKY) with the C-terminal RYamide initially identified in *Aplysia* (145) and also via the identification of ESTs encoding NKY precursors in a variety of mollusks (27). In other respects, sharing a common C-terminal RFamide moiety does not entail that the peptides are functionally or evolutionarily related. Thus, the major challenge will be to get further insight into the evolution and the functional assignment of these various FLPs, especially by focusing on poorly studied mollusk classes. One way of reaching this goal will rely on the characterization of ligand/receptor pairs thanks to the development of methodologies proven efficient in mollusks (87, 104, 118), and in other animal phyla (146–149). Such an approach was efficient to demonstrate the ancestral functional relationship between mollusk LFRFamide and insect sNPF signaling pathways (87). This indicates that sequence homology or disparity alone is not sufficient to ascribe evolutionary relatedness. Within a wide comparative context, the functional characterization of FLP/receptor couples in a variety of bilaterian or eumetozoan species will obviously help define the orthologies between the genes encoding the FLP signaling components. Then, functional and synteny analyses of these orthologous genes might generate unique data to propose convincing scenarios on the evolution of this complex family of neuropeptides in Eumetazoa.

## Conflict of Interest Statement

The authors declare that the research was conducted in the absence of any commercial or financial relationships that could be construed as a potential conflict of interest. The Guest Associate Editor Karine Rousseau declares that, despite being affiliated with the same institution as authors Céline Zatylny-Gaudin and Pascal Favrel, the review process was handled objectively and no conflict of interest exists.
